# A Simplified Classification System for In-Transit Melanoma Metastases

**DOI:** 10.1245/s10434-025-18542-9

**Published:** 2025-11-10

**Authors:** Sofia Breeze, Clare Peterson, Jennifer Garioch, Jenny Nobes, Marc Moncrieff

**Affiliations:** 1 Norwich Medical School, University of East Anglia, Norwich, UK; 2 Department of Plastic and Reconstructive Surgery, Norfolk and Norwich University Hospital, Norwich, UK; 3 Department of Dermatology, Norfolk and Norwich University Hospital, Norwich, UK; 4 Department of Oncology, Norfolk and Norwich University Hospital, Norwich, UK

**Keywords:** In-transit metastases, Melanoma, Classification, Prognosis

## Abstract

**Background:**

In-transit metastases (ITMs) are challenging to treat because of their heterogenous disease course and chronic, relapsing–remitting nature. Specific factors associated with worse prognosis are poorly understood and not included in current American Joint Committee on Cancer classifications. An ITMs-specific classification system to aid treatment decisions and clinical trial design is lacking.

**Methods:**

This study involved 142 patients (M 44%, F 56%; median age 70 years [interquartile range 62–77]) with ITMs from a single, cutaneous melanoma. Baseline melanoma and ITMs characteristics, disease progression, and survival outcomes were collected from a prospective database. The primary outcome was disease-specific survival (DSS). A subgroup analysis excluding stage IV disease at diagnosis was performed.

**Results:**

A longer ITMs-free interval was associated with a longer DSS (hazard ratio [HR] 0.99; 95% confidence interval [CI] 0.98–1.00; *p* = 0.027). A higher number of ITMs and greater Breslow thickness was associated with a shorter DSS (HR 1.25; 95% CI 1.04–1.51; *p* = 0.020 and HR 1.10; 95% CI 1.04–1.17; *p* = 0.001). No independent predictors of DSS were identified. On multivariable analysis, larger ITMs and synchronous regional disease correlated with a worse distant metastasis-free survival (HR 1.02; 95% CI 1.03–1.31; *p* = 0.015 and HR 2.61; 95% CI 1.44–4.72; *p* = 0.002). Maximum threshold analysis selected the optimal cut-point for continuous variables: two lesions for number of ITMs and 30 mm for size at initial diagnosis, 2 mm for primary melanoma Breslow thickness, and 20 months for ITMs-free interval (time from primary melanoma diagnosis to ITMs onset), adjusted to 18 months for clinical relevance.

**Conclusion:**

Patients presenting with more than two and/or >30 mm ITMs at first diagnosis, short ITM-free interval (≤18 months), and synchronous regional disease should be considered at higher risk for disease progression and death.

**Supplementary Information:**

The online version contains supplementary material available at 10.1245/s10434-025-18542-9.

In-transit metastases (ITMs) are a significant problem for patients with melanoma. ITMs develop as macroscopic deposits of tumor disseminated in the lymphovascular system located anywhere between the primary site and the regional draining lymph nodes. The incidence of ITMs is approximately 5–10%, with the risk increasing in direct proportion to the American Joint Committee on Cancer (AJCC) T-stage.^1^ At initial diagnosis of ITMs, patients present with disease burden ranging widely, from an isolated, small lesion with no evidence of metastatic spread to bulky, symptomatic lesions, often combined with synchronous regional and/or distant metastases. The presence of ITMs is associated with a poor prognosis.^2^ In scar recurrences, satellite lesions and ITMs have arbitrary definitions based on the proximity of lesions to the primary tumor site; however, in reality, the prognosis and treatment pathways remain the same regardless, and the term ITMs serves in all instances.

The treatment strategies available for ITMs are broadly classified into ablative and non-ablative, ranging from locoregional to systemic therapies. Often the treatment options offered to a patient depend upon the resources available at the referral center. With the advent of effective systemic therapy strategies for advanced melanoma, a further layer of complexity has now arisen in the decision-making process of sequencing treatments into first-line and second-line options, especially for a low disease burden of ITMs, without evidence of metastases elsewhere. ITMs often have a chronic relapsing–remitting disease course, whereby patients develop lesions that completely resolve with treatment but develop relapses months to years later. In general, patients with a complete response to locoregional therapies, such as isolated limb infusion (ILI), have better overall survival (OS) than those whose disease does not respond or rapidly progresses.^3^ Currently, there are no reliable biomarkers to predict treatment response, further challenging clinicians deciding the first-line treatment strategies for these patients.

Currently, ITMs are classified within stage III of the AJCC melanoma classification system.^4^ This categorizes patients based on the presence or absence of ITMs. It does not subclassify patients based on specific ITMs characteristics. Furthermore, resectable and unresectable disease patterns are often classified in the same AJCC stage III subgroup, yet the treatment pathways are substantially different. The purpose of the AJCC classification system is to stratify patients according to prognosis but, given the diversity in presentation and progression of ITMs specifically, a more clinically relevant classification system is required to enable clinicians to manage their patients appropriately, indicating which patients are more likely to benefit from initial management with locoregional treatment and which require systemic treatment. Furthermore, the patient population in research could be described more accurately, allowing for better comparisons with results of other research and with patients in clinical practice. A classification system that is more precisely able to stratify patients with ITMs based on their disease burden and likely disease course would also facilitate discussions with patients. The aim of this study was to investigate whether readily identifiable and reproducible clinical factors could be incorporated to develop a useful classification system specific to ITMs, to be used as an adjunct to the AJCC melanoma classification system, that could standardize stratification of ITM disease burden and aid clinical decision making.

## Methods

A retrospective, single-center cohort study was conducted. Ethical approval was granted from the UK Health Research Association (IRAS ID: 287430). The data were collated from a supra-regional academic cancer center providing isolated limb infusion and specialized locoregional treatment strategies, in addition to systemic therapies such as immunotherapy, and *BRAF-*mutant targeted therapies in the latter stages of the study period. Data were analyzed from a prospectively maintained database incorporating patients who were diagnosed with ITMs between 2005 and 2021. Patients with ITMs from a single primary cutaneous melanoma were included, whereas those with multiple primary melanomas, non-cutaneous melanoma, unknown primary melanomas, or other synchronous or metachronous malignancies were excluded.

Baseline patient characteristics were recorded, including age at diagnosis of primary melanoma and ITMs, sex, and Eastern Cooperative Oncology Group performance status.^5^ Primary tumor characteristics recorded were date of diagnosis, site, subtype, Breslow thickness, ulceration, mitotic rate, lymphovascular invasion, perineural spread, tumor infiltrating lymphocyte status, mutation status, co-existent nevus, and microsatellites. ITMs factors included date of diagnosis, AJCC stage (8th edition^4^) at the ITM diagnosis, number of clinically evident lesions at first diagnosis of ITMs, ITMs-free interval (months between the date of diagnosis of the primary and first ITM), cumulative number of lesions following ITM diagnosis, maximum clinical diameter of the largest lesion, in millimeters, and the presence of regional nodal and/or distant disease at the time of ITMs diagnosis. The treatments patients received and whether patients received adjuvant systemic therapy was also recorded. Treatment strategies were categorized as local, regional, or systemic, and detailed summary data are provided in Table S1.

## Statistics

Statistical analyses were performed using Jamovi (version 2.4, Sydney, Australia^6^) running the R project statistical computing language (version 4.1, open source: https://cram.r-project.org^7^) and R-Studio (version 1.8; Boston, MA, USA^8^). Comparisons between subgroups were made using the Mann–Whitney U test for continuous variables and chi-squared test for categorical variables. The primary endpoint was disease-specific survival (DSS), mirroring that used for the current AJCC classification system^4^, with important secondary endpoints including distant metastasis-free survival (DMFS) and OS. The endpoints were recorded as the time, in months, from the initial date of ITM diagnosis to the date of the event or censor date (31 December 2024). US Food and Drug Administration criteria were used as definitions of an event for each endpoint.^9^ The survival outcomes were analyzed with the Kaplan–Meier log-rank method.^10–13^ In addition, hazard ratios (HR) were calculated for both univariable and multivariable analysis using Cox’s proportional hazard method,^14^ which included known melanoma prognostic factors. For continuous variables, maximally selected rank statistic (MSRS) analyses were performed using the validated method described by Lausen and Schumacher^15^ to find a statistically significant cut-point to dichotomize continuous variables into clinically relevant subgroups for further analysis. Subgroup analysis of survival was performed using the Kaplan–Meier log-rank method. However, for multivariable Cox’s proportional hazard regression, these data were entered into the model as the original continuous variables, without dichotomy, to avoid bias and loss of power of the variables.^16^ A subgroup analysis excluding patients with AJCC stage IV disease at diagnosis was also performed.

## Results

Data from 142 patients were analyzed in this study. The baseline characteristics of the patients and their primary melanomas are summarized in Table 1. In total, 56.6% of patients were female, and the median age at ITMs diagnosis was 70 years (interquartile range [IQR] 62–77). Most patients had an Eastern Cooperative Oncology Group performance status of 0 or 1 (124/142; 87.3%). The median Breslow thickness was 3.1 mm (IQR 2.1–5.4), and ulceration was present in 47.4%. Sentinel lymph node biopsy had been undertaken to stage the primary melanoma in 89 patients (62.6%), of which 44 (31.0%) cases were positive, with 31 (34.8%) patients at nodal stage (n-stage) 1, 12 (13.5%) at n-stage 2, and one (1.1%) at n-stage 3. A further 63 (44.4%) patients developed clinical regional nodal metastases. The median time from primary diagnosis to ITMs diagnosis (ITMs-free interval) was 17 months (IQR 7–32). At the initial presentation of ITMs, the median number of lesions was one (IQR 1–2). The median maximum recorded diameter was 13 mm (IQR 8–20), and the median cumulative total number of ITMs was six (IQR 3–12). At the time of diagnosis of ITMs, 45.8% (65/142) of patients presented with ITMs in isolation, 35.2% (50/142) had synchronous regional lymph node involvement, and 19.0% (27/142) had synchronous distant disease. The initial treatment strategy used to treat the ITM was local in 107 (75.4%) patients, regional in 17 (12.0%), and systemic in 14 (9.9%), with a total of 85 (59.9%) patients receiving systemic therapy (Table S1). Most patients were treated after 2012 (97/142; 68.3%).

**Table 1:** Baseline characteristics of patients, primary melanoma, and in-transit metastases (ITM) (*n* = 142). (IQR) = interquartile range

Baseline characteristics	
***Patients and primary melanoma***	
*Sex*	
Female	79 (56.6%)
*Age at diagnosis of primary melanoma in years*(median, [IQR])	70 [59–75]
*Site*	
Axial (head, neck or torso)	45 (31.7%)
Extremity (upper or lower limb)	97 (68.3%)
Breslow thickness (median, [IQR])	3.1 [2.1–5.4]
Unknown	5
*Ulceration*	
Present	63 (47.4%)
Unknown	9
*Lymphovascular invasion*	
Present	26 (21.8%)
Unknown	23
*Microsatellites*	
Present	27 (23.9%)
Unknown	29
*Genetic mutations*	
BRAF wildtype	71 (58.7%)
BRAF	46 (38.0%)
NRAS	3 (2.5%)
C-KIT	1 (0.8%)
Unknown	21
***Patients and in-transit disease***	
*Age at ITMs diagnosis, years* (median, [IQR])	70 [62–77]
*AJCC stage at ITMs diagnosis*	
IIIB	32 (22.5%)
IIIC	79 (55.6%)
IIID	4 (2.8%)
IV	27 (19.0%)
*ITMs-free interval, months* (median, [IQR])	17 [7–32]
*ITMs count at first diagnosis* (median, [IQR])	1 [1–2]
*Maximum recorded diameter*, mm (median, [IQR])	13 [8–20]
*Cumulative number of ITMs* (median, [IQR])	6 [3–12]
*Regional lymph node involvement at ITM*	
Present	50 (35.2%)
*Distant disease at ITM*	
Present	27 (19.0%)
*Total number of distant sites involved* (median, [IQR])	1 [0–3]

*AJCC* American joint committee on cancer, 8^th^ edition

**Fig. 1:**
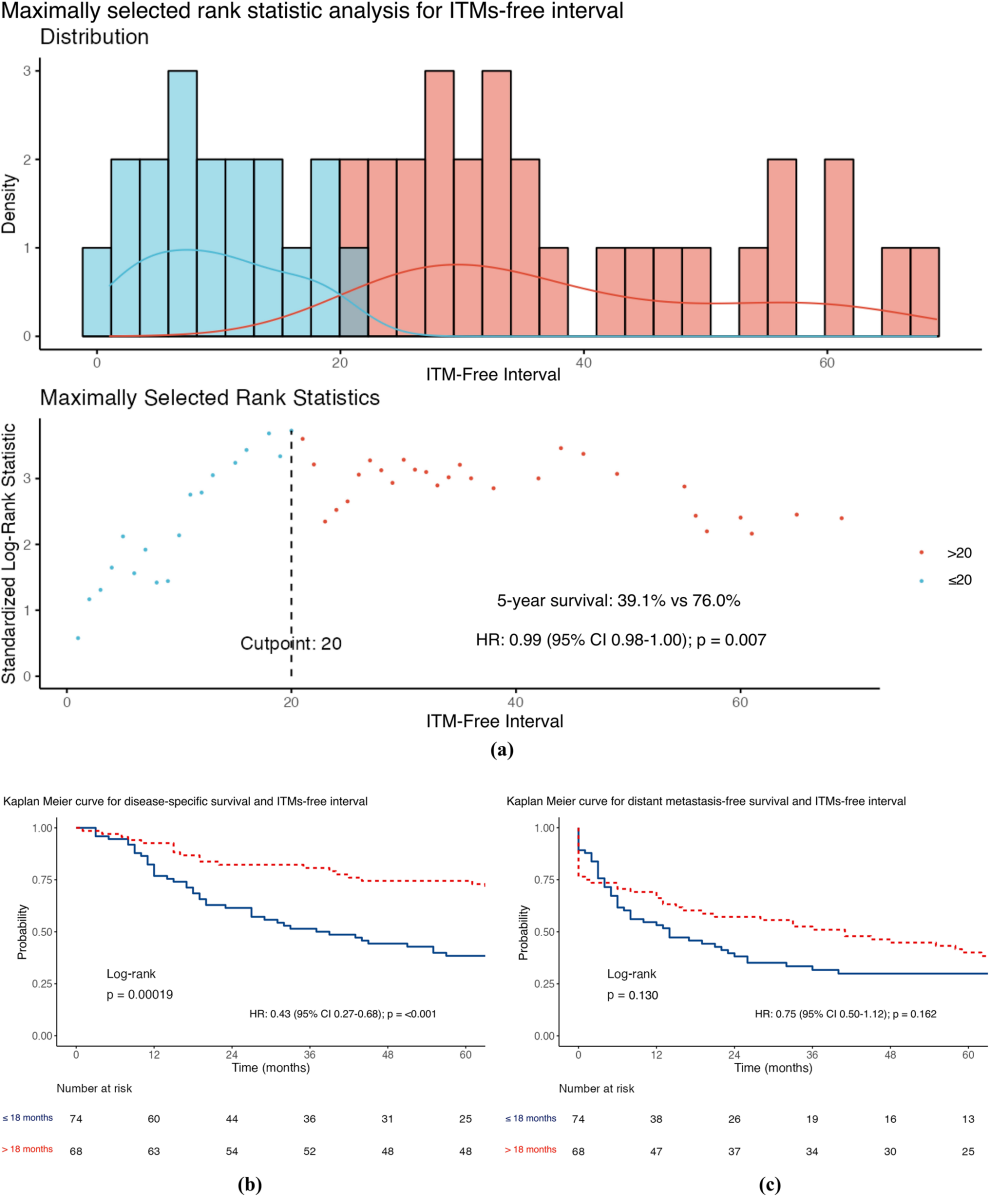

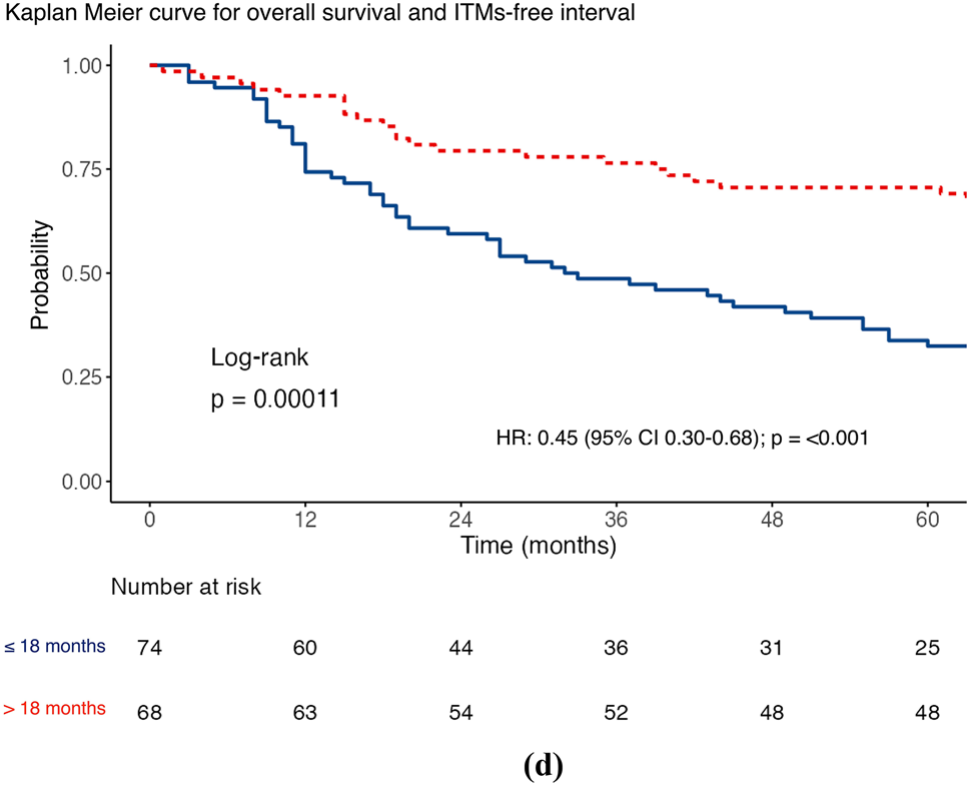
Maximally selected rank statistic analysis for in-transit metastases (ITMs)-free interval using **a** disease-specific survival and Kaplan–Meier curves for ITMs-free interval stratified by the 18 month cut-point for **b** disease-specific survival, **c** distant metastasis-free survival, and **d** overall survival

**Fig. 2:**
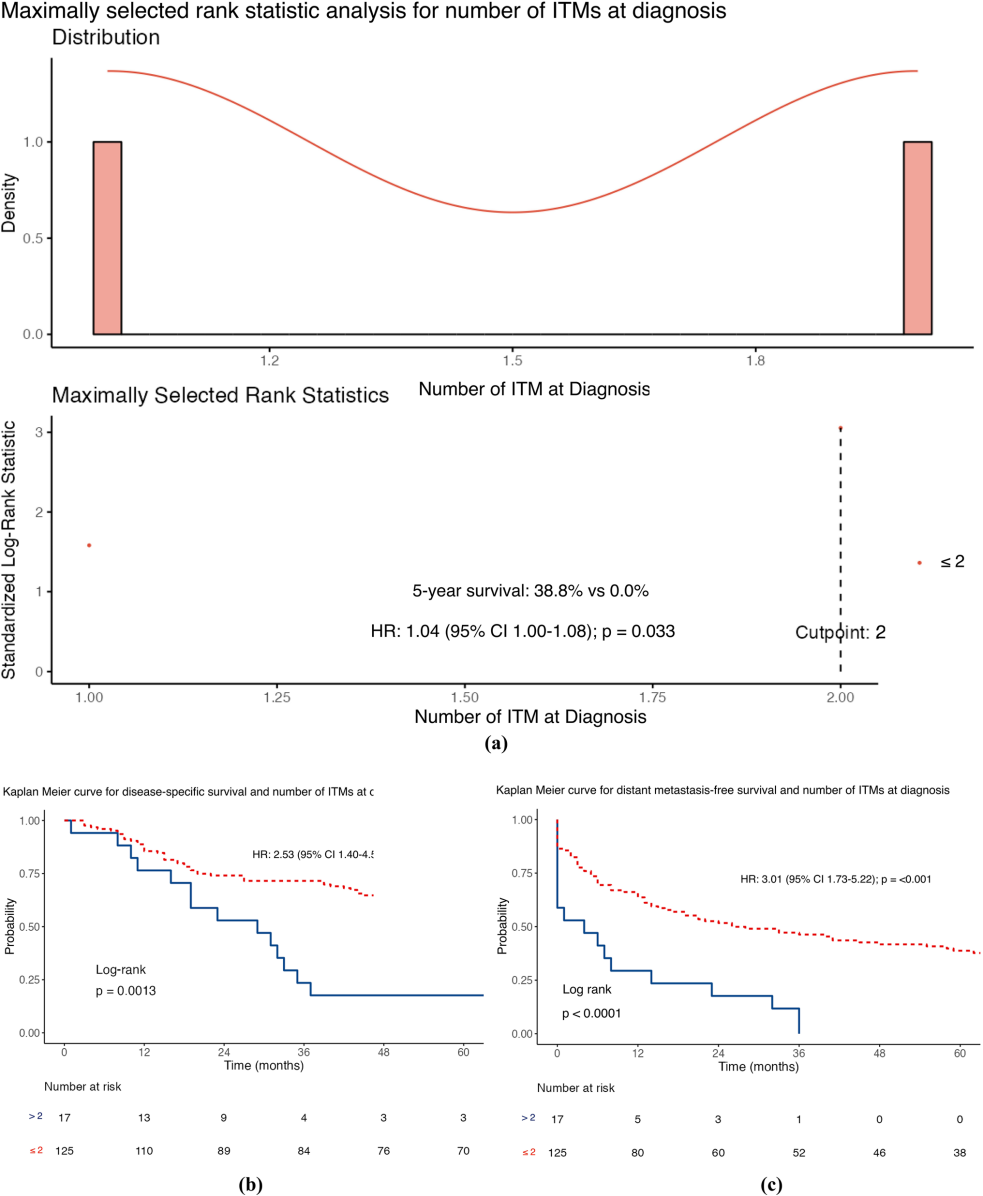

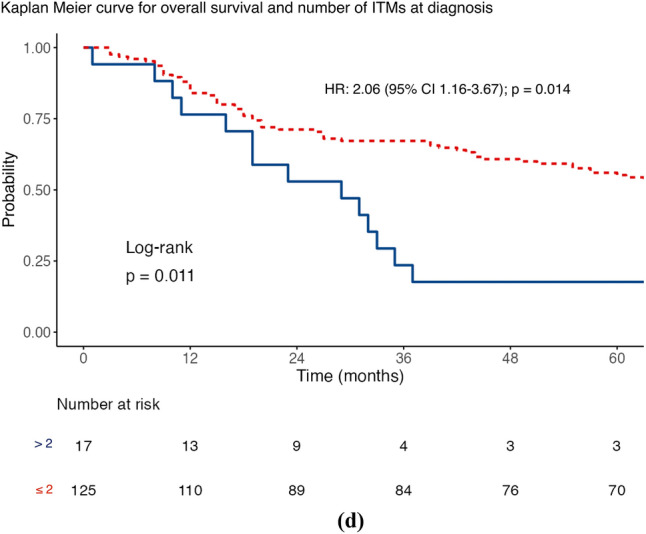
Maximally selected rank statistic analysis for the number of in-transit metastases (ITMs) at diagnosis using **a** distant metastasis-free survival and Kaplan–Meier curves for number of ITMs at diagnosis stratified by more than two and two or fewer lesions for **b** disease-specific survival, **c** distant metastasis-free survival, and **d** overall survival (2d)

**Fig. 3:**
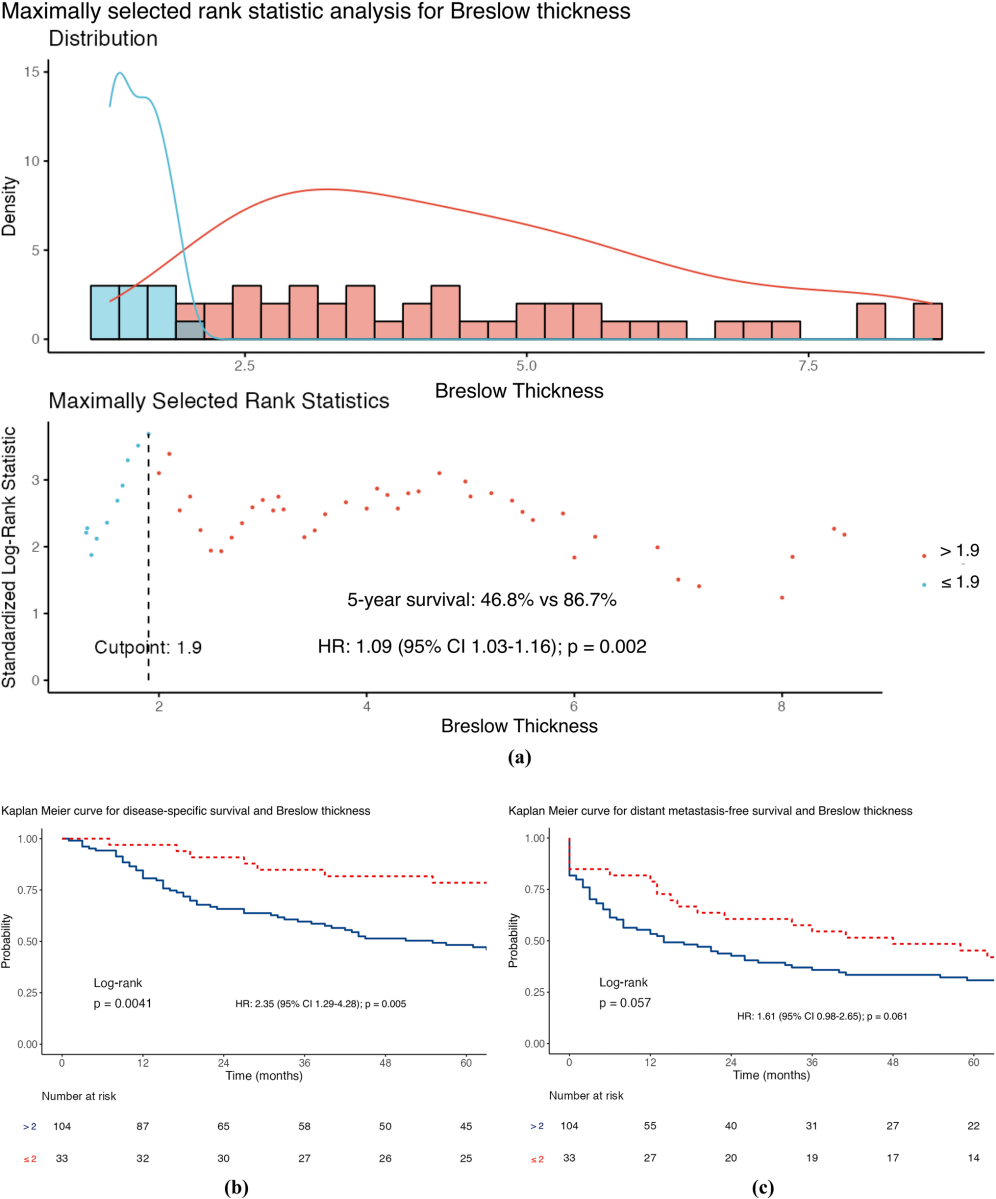

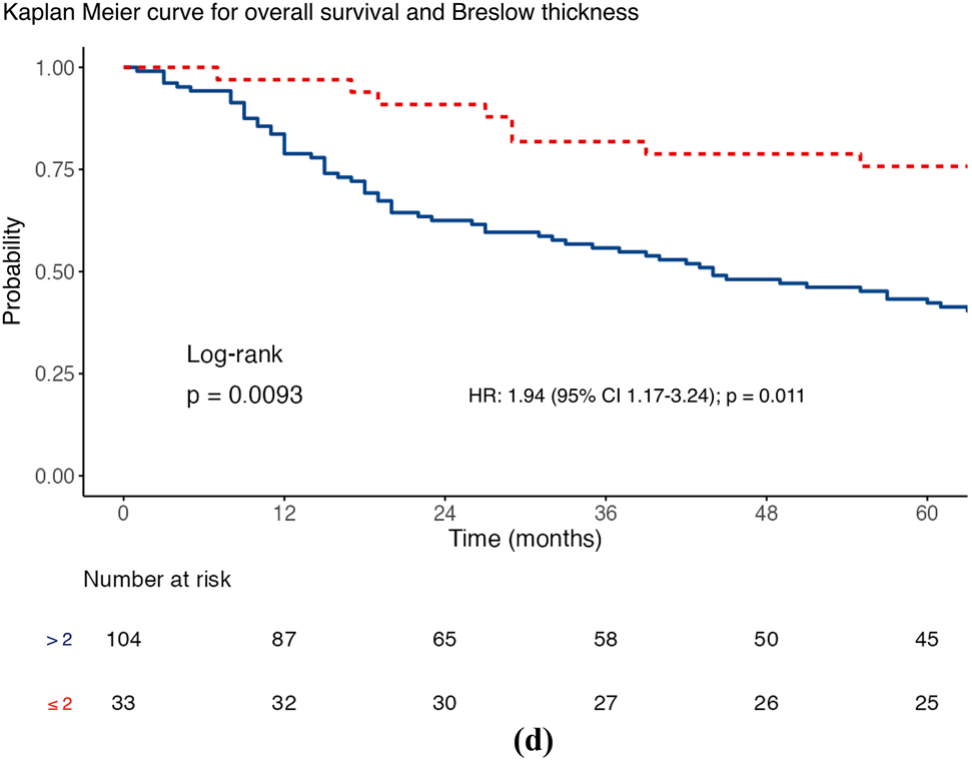
Maximally selected rank statistic analysis for Breslow thickness using **a** disease-specific survival and Kaplan–Meier curves for Breslow thickness stratified by the 2 mm cut-point for **b** disease-specific survival, **c** distant metastasis-free survival, and **d** overall survival

**Fig. 4:**
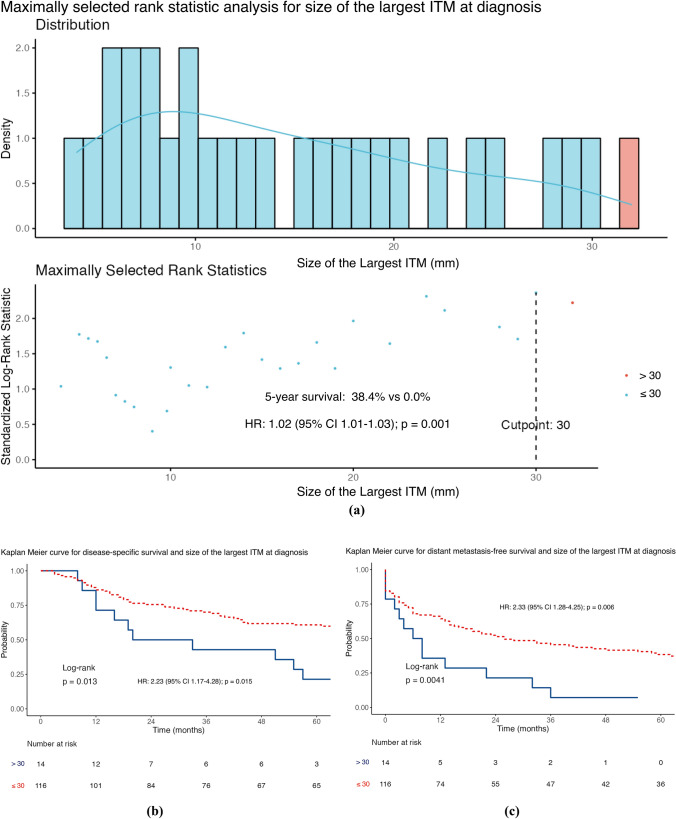

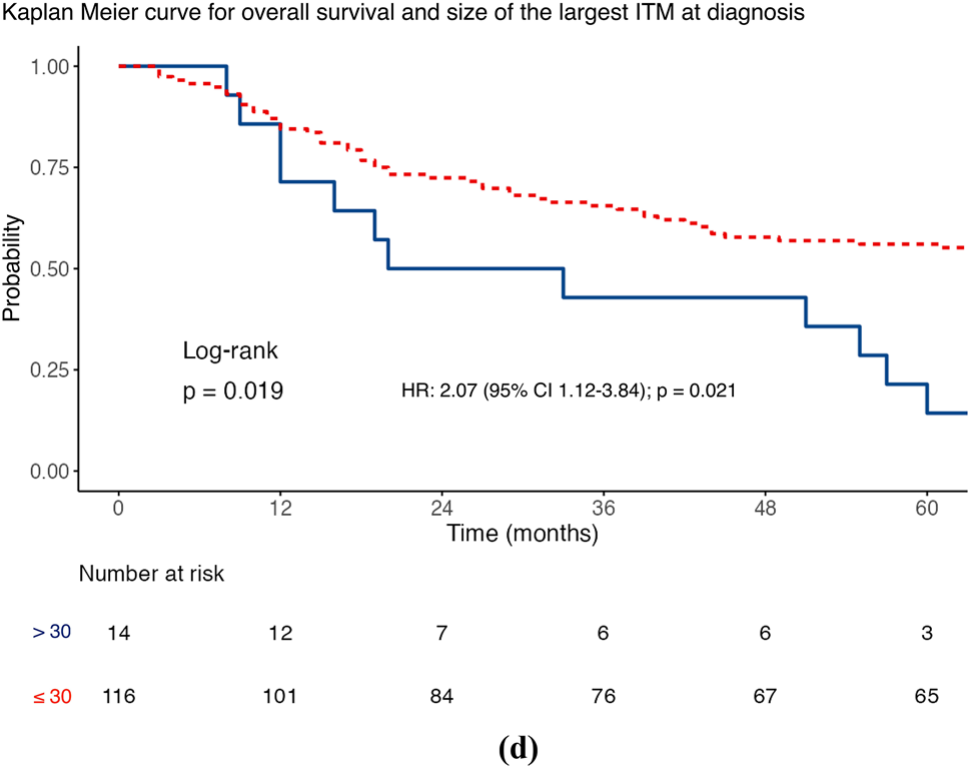
Maximally selected rank statistic analysis for size of the largest in-transit metastases (ITMs) at diagnosis using **a** distant metastasis-free survival and Kaplan–Meier curves stratified by the 30 mm cut-point for **b** disease-specific survival, **c** distant metastasis-free survival, and **d** overall survival

The median DSS was 61 months (IQR 19–104), with 56.3% of patients achieving this (80/142). The factors associated with a worse DSS (Table 2) were the presence of a higher Breslow thickness (HR 1.10; 95% confidence interval [CI] 1.04–1.17; *p* = 0.001) and a higher number of ITMs at first diagnosis (HR 1.25; 95% CI 1.04–1.51; *p* = 0.020) after univariable analysis. However, a longer DSS was associated with an increasing ITMs-free interval (HR 0.99; 95% CI 0.98–1.00; *p* = 0.027). MSRS analyses determined that the optimal cut-point for stratifying ITMs-free interval was 20 months, using the DSS endpoint. Given the standard follow-up period in our practice is 3–6 months, the cut-point was modified to 18 months to be clinically relevant. Furthermore, MSRS analyses with OS as the endpoint highlighted 18 months as a statistically significant cut-point (HR 0.45; 95% CI 0.30–0.68; *p* ≤ 0.001; Fig. 1c). The optimal cut-point for Breslow thickness was 1.9 mm (HR 2.99; 95% CI 1.53–5.85; 0.001), which was rounded to 2 mm to align with the current AJCC T-staging subgroups. Similar results were seen for OS (Table 2) and shown in the Kaplan–Meier curves of Figs. 1, 2, 3, and 4.

**Table 2:** Distant metastasis-free survival, disease-specific survival and overall survival stratified according to sex, site of melanoma, ulceration status, disease burden at in-transit metastases (ITM) diagnosis, adjuvant systemic therapy use, Breslow thickness, age at ITM diagnosis, ITM-free interval, ITM count at diagnosis, and size of the largest ITM at diagnosis (*n* = 142)

Survival endpoint	Disease specific		Distant metastasis–free		Overall	
	Crude risk	Adjusted risk^a^	Crude risk	Adjusted risk^a^	Crude risk	Adjusted risk^a^
*Sex*						
Female	*Referent*	*Referent*	*Referent*	*Referent*	*Referent*	*Referent*
Male	1.59 (0.98–2.58) *p* = 0.059	1.29 (0.70–2.37) *p* = 0.409	**1.85 (1.19–2.87)** ***p* = 0.006**	1.29 (0.74–2.27) *p* = 0.371	1.37 (0.89–2.12) *p* = 0.155	1.07 (0.61–1.86) *p* = 0.822
*Site*						
Axial	*Referent*	*Referent*	*Referent*	*Referent*	*Referent*	*Referent*
Extremity	0.92 (0.56–1.53) *p* = 0.725	1.28 (0.66–2.48) *p* = 0.458	0.66 (0.42–1.03) *p* = 0.068	1.29 (0.74–2.24) *p* = 0.369	0.93 (0.59–1.46) *p* = 0.740	1.15 (0.63–2.10) *p* = 0.640
*Ulceration*						
Absent	*Referent*	*Referent*	*Referent*	*Referent*	*Referent*	*Referent*
Present	1.39 (0.86–2.25) *p* = 0.180	0.97 (0.55–1.68) *p* = 0.904	1.10 (0.71–1.70) *p* = 0.676	1.01 (0.61–1.68) *p* = 0.976	1.40 (0.91–2.17) *p* = 0.128	1.05 (0.63–1.74) *p* = 0.860
*Disease burden*						
Local	*Referent*	*Referent*	*Referent*	*Referent*	*Referent*	*Referent*
Regional	1.14 (0.66–1.98) *p* = 0.640	1.21 (0.64–2.29) *p* = 0.559	**2.45 (1.44–4.18)** ***p* = 0.001**	**2.61 (1.44–4.72)** ***p* = 0.002**	1.07 (0.65–1.77) *p* = 0.782	1.22 (0.69–2.17) *p* = 0.501
Distant	1.75 (0.94–3.25) *p* = 0.079	1.84 (0.87–3.92) *p* = 0.112	**–**	**–**	**1.75 (1.00–3.05)** ***p* = 0.048**	**2.17 (1.10–4.29)** ***p* = 0.026**
*Adjuvant systemic therapy*						
Not given	*Referent*	*Referent*	*Referent*	*Referent*	*Referent*	*Referent*
Given	1.45 (0.87–2.41) *p* = 0.149	1.32 (0.75–2.32) *p* = 0.328	**2.25 (1.38–3.64)** ***p* = 0.001**	1.64 (0.97–2.77) *p* = 0.063	1.28 (0.81–2.00) *p* = 0.288	1.22 (0.74–2.00) *p* = 0.441
Breslow thickness	**1.10 (1.04–1.17)** ***p* = 0.001**	1.06 (0.98–1.14) *p* = 0.150	1.03 (0.97–1.09) *p* = 0.354	0.96 (0.89–1.05) *p* = 0.375	**1.09 (1.03–1.15)** ***p* = 0.002**	1.05 (0.98–1.13) *p* = 0.160
Age at ITM diagnosis	1.02 (0.99–1.04) *p* = 0.145	1.01 (0.99–1.04) *p* = 0.250	0.99 (0.97–1.01) *p* = 0.278	1.01 (0.99–1.03) *p* = 0.250	**1.02 (1.00–1.04)** ***p* = 0.037**	1.02 (1.00–1.05) *p* = 0.054
ITMs-free interval	**0.99 (0.98–1.00)** ***p* = 0.027**	0.99 (0.98–1.00) *p* = 0.094	1.00 (0.99–1.00) *p* = 0.702	1.00 (0.99–1.00) *p* = 0.130	**0.99 (0.99–1.00)** ***p* =** **0.040**	0.99 (0.99–1.00) *p* = 0.125
ITMs Count at Diagnosis	**1.25 (1.04–1.51)** ***p* = 0.020**	1.09 (0.87–1.36) *p* = 0.439	**1.43 (1.16–1.76)** ***p* = 0.001**	0.90 (0.73–1.12) *p* = 0.363	1.19 (0.98–1.45) *p* = 0.074	1.04 (0.84–1.30) *p* = 0.715
Size of the largest ITM at diagnosis (mm)	1.01 (1.00–1.02) *p* = 0.106	1.00 (0.99–1.02) *p* = 0.502	**1.02 (1.01–1.03)** ***p* = 0.002**	1**.02 (1.01–1.03)** ***p* < 0.001**	1.01 (1.00–1.02) *p* = 0.064	1.00 (0.99–1.02) *p* = 0.406

Bold values indicate satistical significance *P* <0.05

Data are presented as hazard ratio (95% confidence interval)

^a^ Variables were examined by Cox’s regression, adjusted for sex, site, ulceration status, disease burden, adjuvant systemic therapy use, Breslow thickness, age at ITM diagnosis, ITM interval, ITM count at diagnosis, and size of the largest ITM at diagnosis

The median DMFS was 17 months (IQR 3–62), with 67.6% of patients triggering this event (96/142). On univariable analysis (Table 2), DMFS was shorter in men than in women (HR 1.85; 95% CI 1.19–2.87]; *p* = 0.006), where regional lymph node involvement was present at first ITMs diagnosis (HR 2.45; 95% CI 1.44–4.18; *p* = 0.001), when adjuvant systemic therapy was used (HR 2.25; 95% CI 1.38–3.64; *p* = 0.001), where there was a higher number of ITMs at first diagnosis (HR 1.43; 95% CI 1.16–1.76; *p* = 0.001), and with a greater size of the largest ITM at diagnosis (HR 1.02; 95% CI 1.01–1.03; *p* = 0.002). Using MSRS analysis, the optimal cut-point for the number of clinically evident ITMs at first diagnosis was two lesions (HR 3.01; 95% CI 1.73–5.22; *p* < 0.001; Fig. 2c), using DMFS as the endpoint. The same analysis identified 30 mm as a statistically significant threshold for stratifying the size of the largest ITM at diagnosis (HR 2.33; 95% CI 1.28–4.25; *p* = 0.006; Fig. 4c). Table S3 shows multivariable analyses for DSS, DMFS, and OS using these cut-points.

Multivariable survival analysis (Table 2), stratified for patient and primary tumor factors, revealed that DMFS was worse in those presenting with synchronous regional disease at the time of ITM (HR 2.61; 95% CI 1.44–4.72; *p* = 0.002) and with a greater size of the largest ITM at diagnosis (HR 1.02; 95% CI 1.01–1.03; *p* < 0.001). There was a near-significant worsening of DMFS with the use of adjuvant systemic therapy (HR 1.64; 95% CI 0.97–2.77; *p* = 0.063). A worse OS was observed in those with synchronous distant disease at first diagnosis of ITMs (HR 2.17; 95% CI 1.10–4.29; *p* = 0.026) and a near-significant worse OS for increasing age at ITM diagnosis (HR 1.02; 95% CI 1.00–1.05; *p* = 0.054). ITMs-free interval, ITMs count at diagnosis, and Breslow thickness was not significant after multivariable analysis for DMFS, DSS, and OS. In a multivariable survival analysis excluding stage IV disease (Table S2), synchronous regional metastases at ITM diagnosis (HR 3.22; 95% CI 1.67–6.21; *p* ≤ 0.001), adjuvant systemic therapy use (HR 2.18; 95% CI 1.19–3.99; *p* = 0.012), ITM-free interval (HR 0.98; 95% CI 0.97–1.00; *p* = 0.011), and size of the largest ITM at diagnosis (HR 1.02; 95% CI 1.01–1.04; *p* = 0.005) were independent predictors of DMFS, and age at ITM diagnosis (HR 1.03; 95% CI 1.01–1.06; *p* = 0.020) was an independent predictor for OS. None were found for DSS.

## Discussion

The diagnosis of ITMs usually heralds a poor prognosis for patients^2^ and provides a significant challenge for clinicians. Many treatment modalities are available, but high-level evidence to suggest which strategies to prioritize at the time of initial presentation are lacking. Fundamental to this lack is the absence of any validated objective measure of the disease burden of the ITMs at presentation, which is anomalous to the rest of the classification system for primary and metastatic melanoma.^4^

Factors used for the primary tumor, such as Breslow depth and ulceration status, in addition to nodal disease burden, further subdivided into micro- and macrometastatic disease, are extensively validated prognostic factors that have endured multiple iterations of the AJCC classification system.^4,17,18^ No such classification system exists for ITMs. Microsatellites are thought to represent clinically occult ITMs and are classified as resected stage IIIB or IIIC disease regardless of sentinel node biopsy status.^19,20^ Accordingly, the treatment strategy for microscopic in-transit disease, with or without a positive sentinel node biopsy, is relatively straightforward, in that adjuvant systemic therapy is routinely offered if the patient’s performance status and comorbidities allow.^21^ Paradoxically, for clinically evident ITMs, the treatment strategies are not well-defined, and high-level comparative research evidence is distinctly lacking.

Creating a tightly delineated, ITMs-specific classification system would aid research and clinical practice. For research, it would allow for stricter eligibility criteria, improved matching of participants, and adjusting for disease severity as a confounding factor, improving the quality of research and accuracy in application to clinical practice. For clinical practice, a classification system would improve prognostic predictions, assisting with decisions about management and aiding communication with patients so they can better understand their condition and prognosis.

Although the current AJCC classification system is based on DSS as the endpoint,^4^ it is important that any classification system for ITMs correlates with DMFS, as this significantly affects the choice of first-line therapy. Our analyses confirm that patients with concurrent locoregional disease at the time of ITMs presentation are at higher risk of early distant relapse and are more likely to be offered systemic therapy; whereas patients with low-disease-burden ITMs, presenting in an indolent fashion, are more likely to be managed with simpler, less toxic local therapies such as intralesional injection,^22,23^ topical therapy,^3^ or surgical ablation. Given that treatment for ITMs can be functionally subdivided into locoregional and systemic strategies, it was important for our analyses to reflect and aid in this decision-making process. The results demonstrated that the significant associations found with DSS, OS, and DMFS can be subdivided into the time of onset of ITMs disease and ITMs disease burden.

### ITM Disease-Free Interval

In their comprehensive analysis of 505 patients at the Melanoma Institute of Australia in Sydney, Read et al.^2^ found that the median interval from primary diagnosis to diagnosis of ITMs was 18 months. The analysis of our cohort mirrored this finding, with a median interval of 17 months. Subsequent MSRS survival analyses identified that a short ITMs-free interval of up to 18 months correlated with worse DSS and OS. Thus, the periodicity of the disease is important, and ITMs that develop rapidly after diagnosis are significantly associated with more aggressive disease on univariable analysis. Accordingly, given that rapid onset of ITMs (<18 months from primary diagnosis) is a significantly poor prognostic sign, we suggest that these patients should be offered first-line systemic therapy, regardless of the disease burden at initial ITMs presentation, to avoid unnecessary toxicity and treatment delay from locoregional therapies, which are more likely to fail early at distant sites (Table 2).

### Disease Burden

Multivariable analysis for DMFS indicated that the prime risk criterion for distant relapse was the presence of regional lymphadenopathy at the time of initial ITMs diagnosis, which would in turn suggest that systemic therapy as first-line therapy is favored in that scenario. This finding is consistent with analysis by Read et al.^2^ where synchronous regional lymphadenopathy at the time of ITMs presentation was associated with a significantly worse 5-year DSS (47% vs 19%; HR 1.82; 1.38–2.40; *p* < 0.001).

An increasing number of ITMs at first diagnosis showed worse DSS and DMFS (Table 2, Fig. 2). Disease burden, defined by the number of ITMs at the time of treatment, has previously been identified as an independent predictor of outcome in a collaborative study by colleagues from Duke and Moffitt Cancer Centers in the USA.^24^ In their analysis, high-risk burden was arbitrarily defined as more than 10 lesions at presentation or any lesion exceeding 2 cm in size. High-risk cases were significantly less likely to respond to ILI, with an overall response rate of 47% compared with 73% in the low-risk group (*p* = 0.002). Notably, disease burden was not associated with OS, possibly because of factors such as the arbitrary nature of the cut-point, referral pathways from other centers, and the historical context of the data collection when effective systemic therapies were not widely available.

A key implication of our analysis, which identified three or more clinically evident lesions and/or lesions >30 mm in size as the cut-point for high-risk disease at the time of initial ITMs presentation, is that the indications for regional perfusion techniques such as ILI may require refinement. Accordingly, ILI is no longer usually offered as first-line therapy to patients with concurrent nodal disease at our center. Similarly, patients with low-burden, low-volume ITMs can be typically managed with first-line ablative or intralesional therapies, which are less resource intensive, carry a lower risk of morbidity, and can often be performed without necessitating referral to a supra-regional cancer center. However, our data also suggest that, where regional perfusion is technically feasible, it remains a viable first-line option in node-negative patients to palliate lesions and avoid the potentially life-altering toxicities of systemic therapy,^25^ particularly in those for whom immunotherapy is contraindicated or in cases of *BRAF* wild-type melanoma. In addition, for axial ITMs or where resources allow, other palliative strategies such as topical diphencyprone^3^ or intralesional injection^23^ may be preferred as initial treatments. The timing of recurrence also warrants consideration, and this locoregional approach may be most appropriate for patients presenting with disease after a prolonged interval, typically more than 18 months following the primary diagnosis.

### Limitations

We acknowledge that the study period spans a time during which both surgical and medical oncology treatment paradigms evolved significantly, including the cessation of routine completion lymph node dissection following a positive sentinel node biopsy and the approval of adjuvant systemic therapy for high-risk stage III disease. As such, the results of analyses involving certain endpoints, particularly DMFS, should be interpreted with caution. This analysis is also based on data from a single center and would benefit from independent external validation. Our institution serves as a supra-regional referral center for the management of ITMs in the East of England, a geographically broad region. However, not all patients diagnosed with ITMs within this area were reviewed through our service, introducing a clear element of selection bias within the cohort.

## Conclusion

We believe our classification system is straightforward to apply and consistent with the current AJCC framework, as disease burden at the time of initial presentation, specifically the presence of more than two lesions, larger ITMs > 30 mm, and/or synchronous regional nodal metastases, are the primary factors used to stratify the risk of relapse or death. Periodicity of the disease is an additional important consideration. We also suggest that this system is clinically relevant to current treatment strategies and may serve as a practical tool to support multidisciplinary decision-making in the management of this complex manifestation of cutaneous melanoma.

## Supplementary Information

Below is the link to the electronic supplementary material.

Supplementary file1 (DOCX 25 kb)
