# Supplementary material for: A Simplified Classification System for In-Transit Melanoma Metastases
Source: Ann Surg Oncol. 2025 Nov 10;33(3):2579–90. doi: 10.1245/s10434-025-18542-9 (PMC12901162; doi:10.1245/s10434-025-18542-9)
Supplement: Supplementary file 1 — Supplementary file1 (DOCX 25 kb) [file 10434_2025_18542_MOESM1_ESM.docx]

**Supplementary Material**

**Table S1:** Table summarising the treatments and the use of adjuvant systemic therapy.

| **Treatment category** | **N = 142** | |
| --- | --- | --- |
| **First line treatment, n (%)**  None  Local^a^  Regional^b^  Systemic^c^ | 4  107  17  14 | (2.8)  (75.4)  (12.0)  (9.9) |
| **Cumulative treatment counts, n (%)^d^**  Local^a^  Regional^b^  Systemic^c^ | 109  91  92 | (76.8)  (64.1)  (64.8) |
| **Treatment strategy used, n (%)**  None  Local only  Regional only  Systemic only  Local and regional  Local and systemic  Regional and systemic  Local, regional and systemic | 4  13  5  15  28  19  9  49 | (2.8)  (9.2)  (3.5)  (10.6)  (19.7)  (13.4)  (6.3)  (34.5) |
| **Adjuvant systemic therapy, n (%)**  No  Yes | 57  85 | (40.1)  (59.9) |
| n, number of participants  ^a^ Wide local excision, ITM excision, CO2 laser ablation, electrochemotherapy, talimogene laherparepvec (T-VEC), Bacillus Calmette–Guérin (BCG), diathermy/fulgurate.  ^b^ Lymph node removal, complete lymph node dissection, diphenylcyclopropenone (DPCP), imiquimod, Isolated Limb Infusion (ILI), amputation.  ^c^ Nivolumab, ipilimumab, ipilimumab/nivolumab, pembrolizumab, dacarbazine, dabrafenib, trametinib, dabrafenib/trametinib, radiotherapy, paclitaxel/trametinib, bevacizumab, paclitaxel, ipilimumab or pembrolizumab, vemurafenib, non-ITM surgical removal, ITM debulking, axilla de-bulking  ^d^Patients received more than 1 treatment so % total is >100. | | |

**Table S2:** Disease-specific survival, distant metastasis-free survival and overall survival stratified according to sex, site of melanoma, ulceration status, disease burden at ITM diagnosis, adjuvant systemic therapy use, Breslow thickness, age at in-transit metastasis diagnosis, in-transit metastasis-free interval, in-transit metastasis count at diagnosis and size of the largest ITM at diagnosis excluding patients with stage IV disease at ITM diagnosis (n=142).

^a^ Variables were examined by Cox’s regression, adjusted for gender, site, ulceration status, disease burden, adjuvant systemic therapy use, Breslow thickness, age at in-transit metastasis diagnosis, in-transit metastasis-free interval, in-transit metastasis count at diagnosis and size of the largest ITM at diagnosis.

| **Survival Endpoint** | **Disease Specific** | | **Distant metastasis-free** | | **Overall** | |
| --- | --- | --- | --- | --- | --- | --- |
|  | **Crude Risk**  HR (95% CIs) | **Adjusted Risk^a^**  HR (95% CIs) | **Crude Risk**  HR (95% CIs) | **Adjusted Risk^a^**  HR (95% CIs) | **Crude Risk**  HR (95% CIs) | **Adjusted Risk^a^**  HR (95% CIs) |
| **Sex** |  |  |  |  |  |  |
| Female | *Referent* | *Referent* | *Referent* | *Referent* | *Referent* | *Referent* |
| Male | **2.06 (1.18-3.59)**  **p=0.011** | 1.83 (0.93-3.60)  p=0.082 | 1.65 (0.98-2.79)  p=0.059 | 1.79 (0.87-3.55)  p=0.117 | 1.51 (0.91-2.49)  p=0.110 | 1.27 (0.68-2.38)  p=0.457 |
| **Site** |  |  |  |  |  |  |
| Axial | *Referent* | *Referent* | *Referent* | *Referent* | *Referent* | *Referent* |
| Extremity | 0.83 (0.46-1.51)  p=0.549 | 1.26 (0.58-2.70)  p=0.561 | 0.75 (0.43-1.31)  p=0.304 | 1.62 (0.77-3.42)  p=0.207 | 0.92 (0.53-1.60)  p=0.774 | 1.15 (0.56-2.36)  p=0.702 |
| **Ulceration** |  |  |  |  |  |  |
| Absent | *Referent* | *Referent* | *Referent* | *Referent* | *Referent* | *Referent* |
| Present | 1.21 (0.70-2.09)  p=0.504 | 0.88 (0.45-1.74)  p=0.716 | 1.06 (0.63-1.80)  p=0.814 | 0.80 (0.42-1.52)  p=0.497 | 1.22 (0.74-2.01)  p=0.437 | 0.82 (0.44-1.53)  p=0.536 |
| **Disease Burden** |  |  |  |  |  |  |
| Local | *Referent* | *Referent* | *Referent* | *Referent* | *Referent* | *Referent* |
| Regional | 1.14 (0.66-1.98)  p=0.639 | 1.30 (0.67-2.51)  p=0.433 | **2.45 (1.44-4.18)**  **p=0.001** | **3.22 (1.67-6.21)**  **p=<0.001** | 1.07 (0.65-1.77)  p=0.785 | 1.39 (0.77–2.51)  p=0.276 |
| **Adjuvant Systemic Therapy** |  |  |  |  |  |  |
| Not given | *Referent* | *Referent* | *Referent* | *Referent* | *Referent* | *Referent* |
| Given | 1.26 (0.72-2.19)  p=0.425 | 1.41 (0.75-2.65)  p=0.289 | **2.04 (1.17-3.58)**  **p=0.012** | **2.18 (1.19-3.99)**  **p=0.012** | 1.09 (0.66-1.79)  p=0.745 | 1.29 (0.73-2.27)  p=0.376 |
| **Breslow Thickness** | **1.10 (1.03-1.17)**  **p=0.005** | 1.07 (0.97-1.17)  p=0.164 | 1.03 (0.96-1.11)  p=0.359 | 0.95 (0.86-1.05)  p=0.297 | **1.09 (1.02-1.15)**  **p=0.008** | 1.06 (0.97-1.15)  p=0.207 |
| **Age at ITM Diagnosis** | 1.02 (1.00-1.05)  p=0.087 | 1.02 (0.99-1.06)  p=0.110 | 1.00 (0.98-1.02)  p=0.926 | 1.02 (0.99-1.04)  p=0.185 | **1.03 (1.01-1.05)**  **p=0.018** | **1.03 (1.01-1.06)**  **p=0.020** |
| **ITMs-Free Interval** | 0.99 (0.98-1.00)  p=0.102 | 0.99 (0.98-1.01)  p=0.338 | 0.99 (0.98-1.00)  p=0.066 | **0.98 (0.97-1.00)**  **p=0.011** | **0.99 (0.98-1.00)**  **p=0.044** | 0.99 (0.98-1.00)  p=0.132 |
| **ITMs Count at Diagnosis**  1  2  3 | *Referent*  0.72 (0.37-1.40)  p=0.337  1.27 (0.45-3.59)  p=0.651 | *Referent*  0.62 (0.29-1.32)  p=0.215  0.73 (0.23-2.37)  p=0.869 | *Referent*  0.94 (0.52-1.72)  p=0.843  1.57 (0.61-4.03)  p=0.350 | *Referent*  0.74 (0.38-1.45)  p=0.384  0.54 (0.19-1.59)  p=0.264 | *Referent*  0.72 (0.40-1.31)  p=0.284  1.03 (0.37-2.87)  p=0.959 | *Referent*  0.73 (0.37-1.42)  p=0.354  0.59 (0.19-1.84)  p=0.364 |
| **Size of the largest ITM at Diagnosis (mm)** | 1.01 (1.00-1.02)  p=0.092 | 1.00 (0.99-1.02)  p=0.869 | **1.03 (1.01-1.04)**  **p=<0.001** | **1.02 (1.01-1.04)**  **p=0.005** | 1.01 (1.00-1.02)  p=0.055 | 1.00 (0.99-1.02)  p=0.673 |
| CIs, Confidence intervals; ITMs, In-transit metastases | | | | | | |

**Table S3:** Disease-specific survival, distant metastasis-free survival and overall survival stratified according to sex, site of melanoma, ulceration status, disease burden at ITM diagnosis, adjuvant systemic therapy use, Breslow thickness (>2 vs ≤2 mm), in-transit metastasis-free interval (>18 vs ≤18 months), in-transit metastasis count at diagnosis (>2 or ≤2), size of the largest ITM at diagnosis (>30 vs ≤30 mm) and age at in-transit metastasis diagnosis (n=142).

| **Survival Endpoint** | **Disease Specific** | | **Distant metastasis-free** | | **Overall** | |
| --- | --- | --- | --- | --- | --- | --- |
|  | **Crude Risk**  HR (95% CIs) | **Adjusted Risk^a^**  HR (95% CIs) | **Crude Risk**  HR (95% CIs) | **Adjusted Risk^a^**  HR (95% CIs) | **Crude Risk**  HR (95% CIs) | **Adjusted Risk^a^**  HR (95% CIs) |
| **Sex Female** | *Referent* | *Referent* | *Referent* | *Referent* | *Referent* | *Referent* |
| Male | 1.56 (0.98-2.58)  p=0.059 | 1.26 (0.69-2.29)  p=0.459 | **1.85 (1.19-2.87)**  **p=0.006** | 1.20 (0.69-2.09)  p=0.525 | 1.37 (0.89-2.12)  p=0.155 | 1.04 (0.60-1.80)  p=0.902 |
| **Site** Axial | *Referent* | *Referent* | *Referent* | *Referent* | *Referent* | *Referent* |
| Extremity | 0.92 (0.56-1.53)  p=0.752 | 1.36 (0.71-2.61)  p=0.353 | 0.66 (0.42-1.03)  p=0.068 | 1.57 (0.88-2.82)  p=0.127 | 0.93 (0.59-1.46)  p=0.740 | 1.27 (0.70-2.30)  p=0.435 |
| **Ulceration** Absent | *Referent* | *Referent* | *Referent* | *Referent* | *Referent* | *Referent* |
| Present | 1.39 (0.86-2.25)  p=0.180 | 0.93 (0.54-1.62)  p=0.809 | 1.10 (0.71-1.70)  p=0.676 | 0.87 (0.53-1.44)  p=0.591 | 1.40 (0.91-2.17)  p=0.128 | 1.00 (0.60-1.65)  p=0.990 |
| **Disease Burden** Local | *Referent* | *Referent* | *Referent* | *Referent* | *Referent* | *Referent* |
| Regional  Distant | 1.14 (0.66-1.98)  p=0.640  1.75 (0.94-3.25)  p=0.079 | 1.09 (0.57-2.08)  p=0.799  1.86 (0.84-4.12)  p=0.129 | **2.45 (1.44-4.18)**  **p=0.001**  - | **3.20 (1.73-5.91)**  **p=<0.001**  - | 1.07 (0.65-1.77)  p=0.782  **1.75 (1.00-3.05)**  **p=0.048** | 1.23 (0.69–2.21)  p=0.481  **2.50 (1.21-5.17)**  **p=0.013** |
| **Adjuvant Systemic Therapy** Not given | *Referent* | *Referent* | *Referent* | *Referent* | *Referent* | *Referent* |
| Given | 1.45 (0.87-2.41)  p=0.149 | 1.46 (0.83-2.55)  p=0.185 | **2.25 (1.38-3.64)**  **p=0.001** | 1.52 (0.89-2.59)  p=0.122 | 1.28 (0.81-2.00)  p=0.288 | 1.32 (0.80-2.18)  p=0.271 |
| **Breslow Thickness** ≤2 mm  >2 mm | *Referent*  **2.58 (1.28-5.22)**  **p=0.008** | *Referent*  1.75 (0.79-3.86)  p=0.168 | *Referent*  1.43 (0.83-2.24)  p=0.195 | *Referent*  0.71 (0.37-1.35)  p=0.290 | *Referent*  **1.97 (1.11-3.51)**  **p=0.021** | *Referent*  1.28 (0.66-2.49)  p=0.471 |
| **ITMs-Free Interval** ≤18 months  >18 months | *Referent*  **0.42 (0.25-0.69)**  **p=0.001** | *Referent*  **0.45 (0.25-0.83)**  **p=0.010** | *Referent*  0.76 (0.49-1.18)  p=0.224 | *Referent*  **0.44 (0.25-0.75)**  **p=0.003** | *Referent*  **0.44 (0.28-0.69)**  **p=<0.001** | *Referent*  **0.44 (0.26-0.76)**  **p=0.003** |
| **ITMs Count at Diagnosis** ≤2  >2 | *Referent*  **2.28 (1.15-4.53)**  **p=0.018** | *Referent*  1.36 (0.62-2.96)  p=0.442 | *Referent*  **2.27 (1.19-4.31)**  **p=0.013** | *Referent*  0.54 (0.25-1.16)  p=0.116 | *Referent*  1.84 (0.94-3.59)  p=0.076 | *Referent*  1.06 (0.50-2.23)  p=0.876 |
| **Size of the largest ITM at Diagnosis (mm)** ≤30 mm  >30 mm | *Referent*  **2.11 (1.10-4.06)**  **p=0.025** | *Referent*  1.44 (0.72-2.90)  p=0.300 | *Referent*  **2.10 (1.15-3.83)**  **p=0.016** | *Referent*  **2.31 (1.15-4.65)**  **p=0.019** | *Referent*  **1.93 (1.04-3.59)**  **p=0.038** | *Referent*  1.34 (0.70-2.58)  p=0.377 |
| **Age at ITM Diagnosis** | 1.02 (0.99-1.04)  p=0.145 | 1.01 (0.98-1.04)  p=0.449 | 0.99 (0.97-1.01)  p=0.278 | 1.01 (0.98-1.03)  p=0.611 | **1.02 (1.00-1.04)**  **p=0.037** | 1.02 (1.00-1.05)  p=0.091 |
| CIs, Confidence intervals; ITMs, In-transit metastases; ^a^ Adjusted using Cox’s regression | | | | | | |
